# Extracellular Electrophysiological Measurements of Cooperative Signals in Astrocytes Populations

**DOI:** 10.3389/fncir.2017.00080

**Published:** 2017-10-23

**Authors:** Ana L. G. Mestre, Pedro M. C. Inácio, Youssef Elamine, Sanaz Asgarifar, Ana S. Lourenço, Maria L. S. Cristiano, Paulo Aguiar, Maria C. R. Medeiros, Inês M. Araújo, João Ventura, Henrique L. Gomes

**Affiliations:** ^1^Faculdade de Ciências e Tecnologia, Universidade do Algarve, Faro, Portugal; ^2^Instituto de Telecomunicações, Lisboa, Portugal; ^3^Departamento de Ciências Biomédicas e Medicina, Universidade do Algarve, Faro, Portugal; ^4^Centro de Investigação em Biomedicina, Universidade do Algarve, Faro, Portugal; ^5^Centro de Ciências do Mar, Universidade do Algarve, Faro, Portugal; ^6^Instituto de Engenharia Biomédica, Universidade do Porto, Porto, Portugal; ^7^Instituto de Investigação e Inovação em Saúde, Universidade do Porto, Porto, Portugal; ^8^Departamento de Engenharia Electrotécnica e de Computadores, Instituto de Telecomunicações, Universidade de Coimbra, Coimbra, Portugal; ^9^Departamento de Física e Astronomia, Instituto de Física dos Materiais da Universidade do Porto, Instituto de Nanociências e Nanotecnologia, Universidade do Porto, Porto, Portugal

**Keywords:** astrocytes, electrophysiology, electrodes, signals, extracellular, glia

## Abstract

Astrocytes are neuroglial cells that exhibit functional electrical properties sensitive to neuronal activity and capable of modulating neurotransmission. Thus, electrophysiological recordings of astroglial activity are very attractive to study the dynamics of glial signaling. This contribution reports on the use of ultra-sensitive planar electrodes combined with low noise and low frequency amplifiers that enable the detection of extracellular signals produced by primary cultures of astrocytes isolated from mouse cerebral cortex. Recorded activity is characterized by spontaneous bursts comprised of discrete signals with pronounced changes on the signal rate and amplitude. Weak and sporadic signals become synchronized and evolve with time to higher amplitude signals with a quasi-periodic behavior, revealing a cooperative signaling process. The methodology presented herewith enables the study of ionic fluctuations of population of cells, complementing the single cells observation by calcium imaging as well as by patch-clamp techniques.

## Introduction

The observation that astroglial cells participate in the control of synaptic transmission in an active manner has been considered a breakthrough in neuroscience. Nowadays astrocytes are recognized to receive signals from neurons, actively responding to neuronal and synaptic activity (Cornell-bell et al., 1990; Poskanzer and Yuste, 2011; Baxter, 2012; Olsen et al., 2015; Bellot-Saez et al., 2017). This was evidenced using calcium imaging (Berridge et al., 2003) and electrophysiological techniques (Bordey and Sontheimer, 1998; Dallérac et al., 2013).

Calcium signaling has proven to be a powerful approach to explore the relevance of glial interactions and has enabled the understanding of the importance of gliotransmission and ionic wave propagation in the regulation of neuronal activity. However, Ca^2+^ elevation in astrocytes is only one facet of the multiple processes at play in the astroglial control of synaptic transmission. For example, patch clamp recordings of neurons and astrocytes coupled to Ca^2+^ imaging performed on cortical primary culture showed glial currents that were not associated with Ca^2+^ signals (Murphy et al., 1993). Recent reports combining intracellular recordings with sodium (Na^+^) imaging showed that astrocytes also generate propagating Na^+^ waves (Bernardinelli et al., 2004; Kirischuk et al., 2007; Bennay et al., 2008; Langer et al., 2012). According to patch-clamp measurements astrocytes are not electrically excitable. However, astrocyte membranes show slow fluctuations and a variety of functional channels (Barres, 1991; Sontheimer, 1994; Verkhratsky and Steinhäuser, 2000). The general view is that the number of elements involved in glial signaling is highly complex and new tools are thus required to study the role of astrocytes in neural circuits and in the brain information processing.

Recently, extracellular planar electrodes known as microelectrode arrays (MEAs) (Spira and Hai, 2013) were also used to measure electrical fluctuations in astrocytes (Fleischer et al., 2015). Astrocytes cultured on MEAs exhibited extracellular voltage fluctuations in a broad frequency spectrum (100–600 Hz) after electrical stimulation. However, MEAs and the associated instrumentation are optimized to measure action potentials. In comparison with an action potential, ionic fluctuations generated by astrocytes are approximately one thousand times weaker and slower. Astrocytes generate electrical fluctuations lasting for several seconds, with amplitudes of a few microvolts. To record these ultra-weak signals, electrodes with low intrinsic thermal noise (ideally below 1 μV) are required. The thermal noise of commercially available electrodes in MEA systems easily reach 10 μV. Therefore, MEAs are not ideally suited to study extracellular activity on astrocytes.

Recently, we reported the recording of ultra-weak signals generated by populations of glioma cells (Rocha et al., 2016). The strategy enabling the observation of these weak signals exploits large capacitive electrodes with a low thermal noise. We have demonstrated that we can record extracellular signals with amplitudes below 1 μV (Medeiros et al., 2016). In this paper, we disclose the potential of this ultra-sensitive electrical method to measure extracellular ionic fluctuations in primary cultures of astrocytes.

This paper begins by a description of the sensing electrodes and their ability to record ultra-week electrical signals. Then, the results section provides an overview of long-term recordings of spontaneous signal patterns. It is shown that the time dependence of signal partners is in line with a progressive synchronization process involving an increasing number of cells engaged in a cooperative process. A possible relation with Ca^2+^ signaling is explored by looking at the effects of calcium deprivation on the signal rate and intensity. Finally, the results are discussed and summarized in the context of our present understanding gathered from calcium image and patch-clamp methods.

## Materials and Methods

### Gold Microelectrodes

**FIGURE 1:**
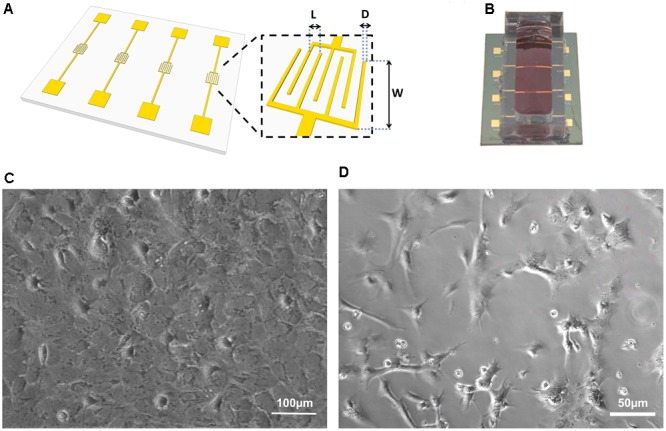
Sensing devices and cells used in this study. **(A)** Schematic diagram of an interdigitated electrode fabricated on a silicon wafer. Device dimensions are *W* = 1000 μm, *L* = 20 μm, and *D* = 15 μm. The total number of fingers is 10. **(B)** Schematic diagram of the device mounted in a vessel with electrical connections. **(C)** and **(D)** are photographs of astrocytes populations. The estimated cell confluence is 90% for the cells in **(C)** and 70% for the cells in photograph **(D)**. These images were recorded using phase contrast microcopy method.

The sensing electrodes used consist of two co-planar, parallel gold tracks on the upper surface of a thermal oxidized silicon wafer. Gold electrodes were deposited by thermal evaporation. The electrode shapes and dimensions are according to the labels in **Figure 1A**, where *W* is the electrode length, *L* the inter-electrode distance, and *D* the electrode depth. Each microelectrode has a total number of 10 interdigitated fingers and each finger has a length of 1000 μm. The total electrode length is 10x*W* = 10.000 μm, *L* = 20 μm, and *D* = 15 μm. The total active sensing area is 150.000 μm^2^. These devices were provided by PHILIPS Research labs in Eindhoven (Netherlands). On top of the interdigitated electrodes a PMMA compartment is glued that can be filled with cells and culture medium. The well is loosely covered with a lid to prevent evaporation of the medium. The system assures the presence of enough cell culture medium to keep the cells viable over more than 24 h without medium change. **Figure 1A** shows a schematic diagram of the electrode and **Figure 1B** shows a photograph of the complete sensing device.

### Animals

C57Bl6/J mice were kept in our animal facility, with controlled temperature (21 ± 1°C) and humidity (55%), with food and water *ad libitum* in a 12 h dark/light cycle. The experiments were performed in accordance with institutional and European guidelines (2010/63/EU) for the care and use of laboratory animals. Both the Portuguese law (DL 113/2013) and the European law (directive 2010/63/EU) state that obtaining tissue for cell cultures without actually performing any procedures in a laboratory animal, as is the case in this paper, does not require an official approval from the competent authority (Direcção Geral de Alimentação e Veterinária, DGAV), since no procedures are performed (the law understands that a procedure is the equivalent of provoking discomfort in an animal similar to a needle piercing the skin), only that the process of sacrificing animals is performed by a licensed user. The animals were kept in our licensed animal house facility. We state that this study was performed according to the guidelines established by our institute, and by law.

### Primary Astrocyte Cultures

Primary mixed glial cultures were obtained from new-born C57Bl6/J mice with 0–3 days (Carreira et al., 2014). Briefly, after decapitation the brains were removed and the meninges and cerebellum were discarded. Brain tissue was then mechanically dissociated and enzymatically digested (0.1% trypsin and 0.001% DNase I, 20 min at 37°C). Cells were seeded in 25 or 75 cm^2^ flasks coated with poly-L-lysine, at a density of 0.2 × 10^6^ cells/cm^2^, and cultured in D-MEM/F12 with GlutaMAX-I supplemented with 10% fetal bovine serum, 0.25% gentamicin and 0.25 ng/ml M-CSF, at 37°C and 95% air/5% CO_2_, in a humidified incubator. Culture medium was replaced every 4 days and confluency was achieved after 15 days, *in vitro*. Microglia and oligodendrocyte precursor cells were removed by vigorous shaking, affording an astrocytes culture with around 98% of purity. After detachment of microglia cells, astrocytes were trypsinized (0.25%, 20 min at 37°C) and seeded on the electronic devices. The cell culture medium used during electrical measurements is the same as used for culturing the cells. An aliquot of 200.000 cells per cm^2^ was transferred to the well and was placed in an incubator (Thermo Scientific, Midi 40). Prior to cell deposition, the micro-structured electrodes were sterilized by UV treatment and the electrodes were coated with poly-L-lysine to promote cell adhesion. The cells were maintained in an incubator at 37°C, keeping a humidified atmosphere with 5% of CO_2_. The system assures the presence of enough cell culture medium to keep the cells viable over more than 24 h without medium change. Cell numbers and viability was assessed using a Neubauer chamber-based trypan blue live/dead exclusion assay. Images of the astrocytes cultures were recorded using a phase contrast microscopy method using an Axiovert 40 CFL microscope from Zeiss. Photographs of the astrocytes populations are shown in **Figures 1C,D**.

### Electrical Measurements

The entire experimental setup was specifically designed for ultrasensitive detection. External interference was minimized through the use of a Faraday cage and low-noise cables. Extracellular voltage measurements were carried out using a low-noise voltage amplifier (SR 560, Stanford Research) and a dynamic signal analyser (35670A, Agilent). To minimize drift, the current amplifier is calibrated and the setup is stabilized for at least 2 h before measuring. The current was recorded as a function of time by using zero bias on the electrodes. Small-signal impedance measurements were carried out using a RCL meter Fluke PM 6306.

The extracellular selective calcium chelator (EGTA) experiment was repeated in three experiments, and the assessment of the resulting difference in the EGTA experiments was done using unpaired student *t*-test. The difference was accepted to be significant with α = 0.05.

## Results

**FIGURE 2:**
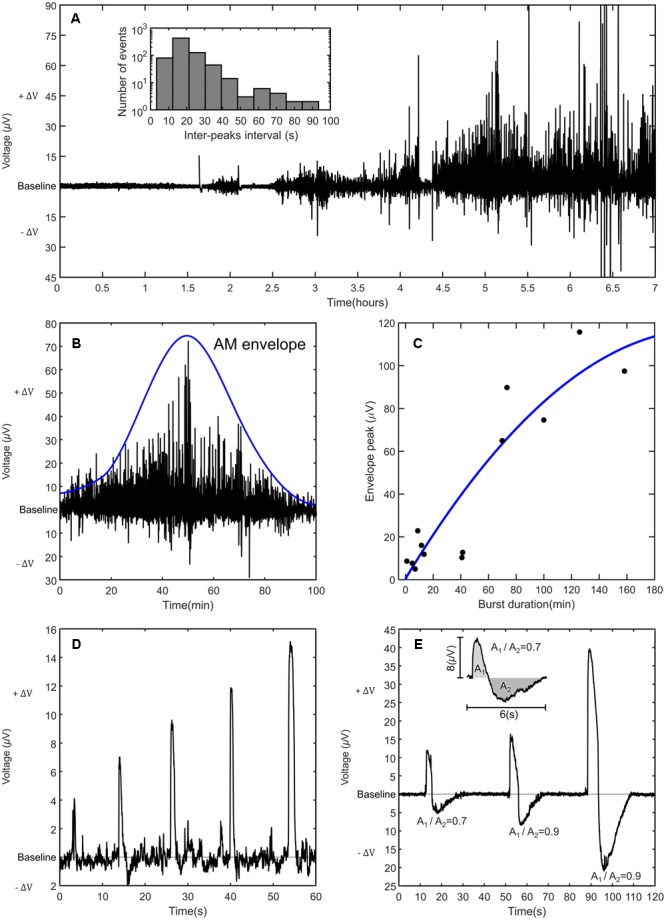
Electrical signals recorded in astrocytes populations. **(A)** An overview of a long-term recording of astrocyte population activity with different amplitude modulated (AM) bursts of activity. The inset shows a histogram of the inter-spike intervals recorded for the whole burst activity shown in **(A)**. **(B)** Typical AM modulated burst. **(C)** Relation between the burst duration and the envelope amplitude. **(D)** Detailed view of several individual signals. **(E)** Asymmetric signals with increasing magnitude. As the signal amplitude increases, the ratio between the areas under positive and negative voltage fluctuations becomes 0.9.

**Figure 2A** shows a typical time trace of astrocytes spontaneous activity. After 1–2 h of cell seeding on the device, astrocytes exhibit spontaneous weak signals. These early stage signals are approximately 1.5 μV in amplitude and they appear in clusters of a few (<10) discrete signals. With time, the astrocyte overall activity evolves to a new type of spontaneous activity, which is characterized by bursts of quasi-periodic signals and an increase of the signal magnitude. These bursts are briefly interrupted by shorter periods where both the signal rate and magnitude are lower. Bursts are characterized by a slow rise in signal amplitude until a maximum is reached. After attaining the maximum, the pattern decays following time dependence similar to the one followed during the rise in amplitude. This behavior originates symmetric signal patterns modulated in amplitude. We refer to these patterns as amplitude modulated (AM) bursts. Several AM burst are visible on **Figure 2A**. The temporal duration of an AM burst may vary from several minutes to hours.

Signals are approximately equidistant, with an inter-peak interval between 3 and 95 s. In order to identify firing patterns, we analyzed the entire time bursting region of **Figure 2A** and constructed the statistics of the time between two consecutive spikes. These inter-peak intervals were analyzed. The mean inter-peak interval (± standard error of the mean) is 16.92 ± 10.4 s. The skewness and kurtosis of the distribution appear significantly larger than their normal distribution thresholds. To evidence this, in the inset of **Figure 2A** we show the histogram obtained by distributing the time intervals into 10 s wide bins. Time intervals shorter than 3 s and longer than 95 s were not considered. The dominating frequency (*f*) is approximately 0.07 Hz (*f* = 1/14 s).

**Figure 2B** shows a typical AM modulated burst. A burst can be characterized by its duration and by its peak in amplitude. The burst is fitted with a smooth curve that outlines the extremes. This is called the AM envelope. From this envelope we estimate the AM envelope peak. **Figure 2B** shows an AM burst of spontaneous activity with duration of 100 min. The estimated AM envelope peak is 74.5 μV. **Figure 2C** shows the relation between the burst duration and the burst amplitude. Long bursts have higher signal amplitudes. For short times the relation is approximately linear. However, for very long times (*t* > 2 h) the fitting curve gently bends, suggesting that this signal amplitude will saturate.

An AM burst is comprised of discrete signals, as the ones shown in **Figure 2D**. With time, the signals increase in amplitude without significant changes in the signal duration (1–2 s). An AM burst may also be comprised of biphasic signals, as the ones represented in **Figure 2E**. The signal shape is asymmetric and characterized by a fast rise time to a peak value. Once the peak is reached the voltage decays, first slowly and then rapidly, to a minimum value lower than the baseline potential. This negative part of the signal relaxes slowly to the baseline.

The signals must be related to the inward and outward flow of ions across the membrane. The positive variation (+ΔV) in respect to the baseline must correspond to an outward flow of positive ions and the negative variation (-ΔV) to a net inward flow of positive ions.

The biphasic signal is clearly asymmetric because the upper and lower voltage peaks are different. However, if the areas under the voltage fluctuations are compared, this asymmetry in relation to the baseline is not so pronounced. For weak signals, the ratio between areas is approximately 0.7. For strong signals, this ratio is 0.9. Furthermore, there is evidence that this ratio is still under-estimated because the positive variation does not have time to relax to the baseline, becoming slightly super-imposed in the negative variation. To some extent, both voltage fluctuations are mixed, and thus the area under the positive fluctuation is somewhat underestimated. It is reasonable to assume that the amount of charge involved in the positive voltage variation is equal to the amount of charge involved in the negative voltage fluctuation. Because the positive voltage variation occurs in a shorter time than the negative fluctuation, we propose that the apparent asymmetry is a consequence of a different ion flow rate.

The NCX channel operating in reverse mode is an obvious candidate to explain the positive voltage fluctuation (net positive outward current). However, further studies are required to elucidate the full signal shape.

A large area electrode cannot resolve single cell signals; the recorded trace reflects the combined activity of an astrocytic ensemble. If a large number of individual cells generate independent signals obeying to the same statistics then, according to the central limit theorem, the resulting activity resembles noise since the distribution of the average of a large number of independent, identically distributed variables will be approximately normal. To explain the observation of discrete, temporally structured signals, several hypotheses may be considered. An obvious one is that the population of cells is synchronized by a biological signaling process (pacemaker mechanism, just as in a cardiac beating signal, where individual signals sum up into an averaged discrete signal). However, a synchronized population of cells is not expected to generate modulating patterns. To explain the existence of AM- or FM-like modulated patterns we need to consider the existence of sub-populations of cells. A sub-population of cells is seen here as a relatively small number of cells that are synchronized. For the following reasoning a sub-population, in the limit, can be a single cell. These sub-populations may be sparse and distributed over the entire electrode area and generate signals with slightly different frequencies and phases, giving rise to patterns with an AM- and FM-like modulation.

**FIGURE 3:**
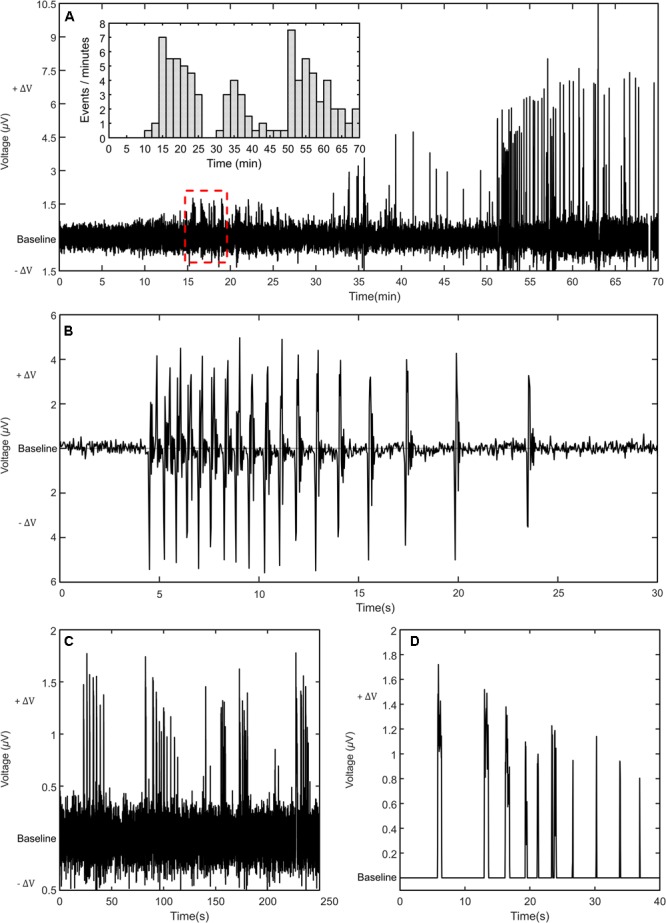
Signal patterns with frequency modulation (FM). **(A)** Time trace showing several distinct bursts. The bar plot in the inset shows that the signal rate decreases with time (FM). **(B)** Typical FM modulation recorded in a cluster of relatively fast signals. **(C)** Detailed view of the clusters of signals inside the dashed square of **(A)**. **(D)** Detailed view of an individual cluster with the noise removed to highlight the changes with time in signal width.

**Figure 3A** shows an example of a second type of burst also frequently recorded during our experiments. These bursts are characterized by a frequency modulation (FM). At the onset of the burst, the frequency rises fast up to 15 spikes/min and then begins to decrease slowly until the signals disappear. The decrease in frequency can vary from a few minutes up to a half-hour. The progressively frequency shift to lower frequencies (red shift) is not necessary accompanied by a decrease in signal amplitude, as observed in the AM modulation discussed above. The FM modulation is clearly observed in **Figure 3A** for *t* > 50 min. Interestingly, when all the clusters in **Figure 3A** are analyzed and the number of spikes per minute is represented in the form of a bar plot, all signal clusters in this time trace exhibit a similar red shift in frequency. **Figure 3B** shows an example of a FM modulation recorded in a cluster of signals.

Calcium oscillations with AM and FM type of modulation have been reported by others (Micali et al., 2015; Balaji et al., 2017). However, in their studies signal modulation appears as a response to a chemical stimulus and it is usually reported in independent time traces (Pasti et al., 1997; Di Capite et al., 2009). To the best of our knowledge, we show for the first time non-interrupted experimental time traces with a clear FM modulation. Furthermore, this frequency shift repeats itself in few consecutives bursts, is spontaneous and is recorded using extracellular electrodes.

In order to highlight the electrical sensitivity of our sensing electrodes and method, we showed in **Figure 3C** a detailed view of the weakest signals measured on the time trace of **Figure 3A**. One cluster is also shown in detail after the noise was removed for clarity (**Figure 3D**). The signals are monophasic and approximately 1.5 μV in amplitude. In the beginning of the cluster the signals are about 1 s long but get shorter and weaker until the cluster fades away.

**FIGURE 4:**
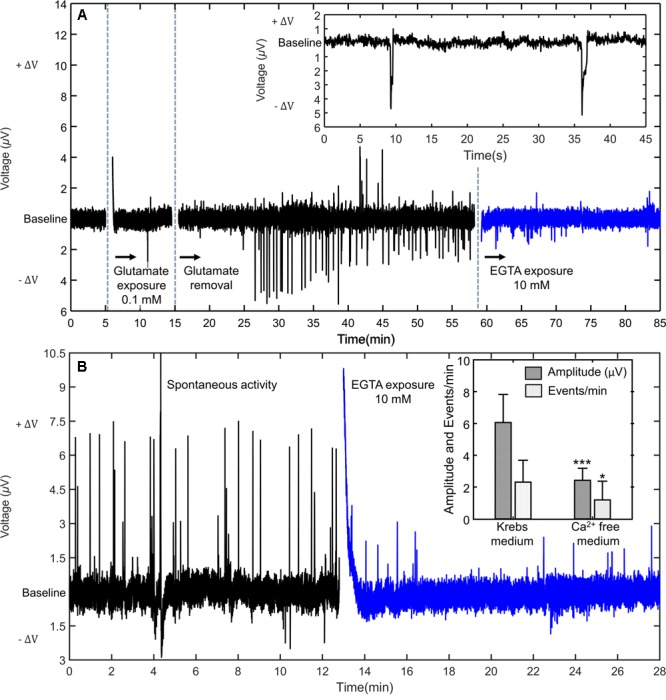
Signal-enhancing and signal-inhibiting agents. **(A)** Typical example of the effect of glutamate exposure. Signals appear with a delay of approximately 20 min. The induced signals are inhibited by an extracellular selective calcium chelator (EGTA). The inset shows a detailed view of two signals. **(B)** The effect of calcium deprivation in a burst of spontaneous signals. The inset shows the average changes, plotted as mean ± SD, caused in the signal rate and signal amplitude, compared to the baseline activity. ^∗^*p* < 0.05; ^∗∗∗^*p* < 0.001.

In order to gain insight into the possible biological process connected to the signals, the astrocyte population was exposed to glutamate (0.1 mM) for 10 min and then removed. In a time span of 20 min after glutamate removal, discrete signals were induced. The effect of glutamate exposure on the astrocyte activity is shown in **Figure 4A**. The inset shows a detailed view of two discrete signals inside the burst of activity.

The signals triggered upon glutamate exposure are monophasic, with a negative voltage fluctuation in relation to the baseline. A detailed view of two signals is shown in the inset of **Figure 4A**. In this respect they are slightly different from the spontaneous signals, which can be monophasic or biphasic.

As explained in the Introduction section, most of the electrical fluctuations reported for astrocytes populations have been directly related with Ca^2+^ signaling. To assess the possible involvement of calcium on our signals we deliberately deprived the cell culture medium of calcium cations and looked at the possible effects on the signals. **Figure 4A** shows that the signals can be substantially inhibited when the cell-surrounding medium is replaced by calcium deprived medium, through the addition of a 10 mM solution of ethylene glycol-bis (β-aminoethyl ether)-N,N,N′,N′-tetraacetic acid (EGTA), a calcium-chelating agent.

**Figure 4B** shows the effect of EGTA exposure in a burst of spontaneous signals. The addition of EGTA also successfully stops astrocyte spontaneous activity. The bar plot in the inset of **Figure 4** shows that in the presence of the calcium-chelating agent, both the average spike rate and the signal amplitude are lowered. The frequency of signals decreases from an average value of 3 signals per minute to only 1 signal per minute. The average amplitude of the signals lowers from approximately 6 μV to 1 μV.

Cells were kept in a medium with EGTA for 15 min, and then the electrolyte medium was replaced with fresh and normal cell culture medium. After removal of the EGTA the astrocyte population remained silent, with occasional signals. One hour after the EGTA removal the astrocyte population re-started activity. This pattern of behavior was observed in all experiments (*n* = 3), with EGTA addition substantially reducing the activity (*p* < 0.001).

The addition of 10 mM of EGTA was conducted to inspect a possible relation between the observed signals and extracellular calcium waves commonly reported for astrocytes. However, it has also been reported that the removal of calcium from the cell culture medium causes a disruption of the connections between cells (tight junctions) (Rothen-Rutishauser et al., 2002). These perturbations may also affect the signaling process.

## Discussion

Astrocytes are interconnected through pores, called gap junctions that enable passage of ions and various small molecules, hence implying ionic and metabolic coupling between cells. This communication path between cells allows them to get synchronized. Optical imaging methods have shown that confluent astrocytes populations get involved into long range extracellular Ca^2+^ waves (Cornell-Bell et al., 1990) and possibly also into Na^+^ waves. The reported optical signals have striking similarities with the electrical signals presented and discussed in this work, namely on signal width, shape and frequency. For example, a study by Kuga et al., 2011 reports the observation of repetitive signals with the periodicity of 1 min and lasting for approximately 10–20 s (similar to the ones in **Figure 2D**). Other experiments (Zur Nieden, 2005) using rat hippocampal astrocytes show spontaneous calcium transients with a frequency of approximately 1.3 signals per minute. Each individual Ca^2+^ signal lasts for approximately 10–20 s. A recent study (Srinivasan et al., 2015) using state-of-the-art image techniques, performed in brain slices and *in vivo*, showed that astrocytes Ca^2+^ fluctuations lasted for approximately 14 s. The reported signals are also characterized by a fast rise time, followed by a slower decay to the baseline.

This resemblance between optical and electrical signals supports the view that the extracellular electrophysiological measurements shown here are capturing the features of some of the well-known astrocytes oscillations.

Having established this correlation between optical and electrical measurements, it is important to evaluate the advantages/disadvantages of each method. Comparatively, optical methods require incubation with fluorescence dyes. Extracellular electrophysiological measurements are long-term, non-invasive and offer a higher sensitivity. However, electrical signals are not selective to ionic species.

This study has demonstrated that extracellular electrophysiological measurements show a richness of detail never reported before using imaging methods. For example, electrical signals rarely start abruptly. Often, a signal pattern is preceded by a sudden increase in noise. Once the signals start, they gently increase in amplitude and rate, showing that an increasing number of cells get synchronized or are somehow correlated. The temporal dependence of the cells synchronization process can be followed in real time. This is a feature not entirely perceived using fluorescence techniques. In imaging techniques the signal-to-noise ratio (SNR) is relatively low. For example, with voltage sensitive dyes, an alternative to calcium imaging which potentially interferes less with the cellular intrinsic dynamics, the fractional change in fluorescence is in the order of 0.1% to 0.5%. The SNR associated to the signals presented here depends on the signal amplitude. However, SNR higher than 30 can be obtained if strong signals as the ones shown in **Figure 2E** are generated.

Extracellular electrical recordings can be performed over periods as long as a week, this enabling the observation of signals patterns that could not be accurately recorded using conventional imaging methods. We have shown signal patterns with FM and AM modulation that repeat in a quasi-periodic trend. These modulations are known, and have been proposed as a way to encode biological information (Berridge et al., 2003). Previous studies have shown that the modulations occur in response to chemical stimuli (Pasti et al., 1997) and have been recorded in distinct time traces. To the best of our knowledge, this work provides the first report on FM and AM signal modulations occurring spontaneously, monitored in real time in a single time trace.

## Conclusion

In summary, our work yielded a new methodology to measure extracellular signals in astrocytes populations. This experimental approach enables the study of astrocyte physiology in a totally non-invasive manner, with a high temporal resolution, and over extended periods of time. The advantages of the method were demonstrated by showing a variety of spontaneous signals, in close agreement with previous reports using optical fluorescence methods. Furthermore, newly revealed signals and signal patterns, which had remained inaccessible using conventional imaging and patch-clamp methods, have also emerged.

We believe the methodology used here can be applied to brain slices and to mixed cell populations to study neuron-glia interactions. This is because, while action potentials are relatively fast signals (milliseconds) detected in a frequency range of kHz, signals from astrocytes populations are observed in a spectral region below 10 Hz. Therefore, both types of signals should not interfere with each other.

It is proposed that extracellular measurements in astrocytes, complemented with other methods, will hopefully produce a more detailed knowledge of astrocytes signaling, paving the way towards a better understanding of the brain information processing.

## Author Contributions

AM prepared the cell cultures and carried out the electrical measurements. PI automated the recordings and wrote the software for the data analysis. SA, PI, and YE performed the electrical measurements for all figures. AL and IA extracted the cells from new-born mice. MC suggested and supervised the addition of EGTA. PA, MM, JV, MC and HG wrote the manuscript. HG supervised the project.

## Conflict of Interest Statement

The authors declare that the research was conducted in the absence of any commercial or financial relationships that could be construed as a potential conflict of interest.

## References

[B1] Balaji R. Bielmeier C. Harz H. Bates J. Stadler C. Hildebrand A. (2017). Calcium spikes, waves and oscillations in a large, patterned epithelial tissue. *Sci. Rep.* 7:42786. 10.1038/srep42786 28218282 PMC5317010

[B2] Barres B. A. (1991). Glial ion channels. *Curr. Opin. Neurobiol.* 1 354–359. 10.1016/0959-4388(91)90052-9 1726551

[B3] Baxter P. (2012). Astrocytes: more than just glue. *Dev. Med. Child Neurol.* 54 291–291. 10.1111/j.1469-8749.2012.04232.x 22404619

[B4] Bellot-Saez A. Kékesi O. Morley J. W. Buskila Y. (2017). Astrocytic modulation of neuronal excitability through K^+^ spatial buffering. *Neurosci. Biobehav. Rev.* 77 87–97. 10.1016/j.neubiorev.2017.03.002 28279812

[B5] Bennay M. Langer J. Meier S. D. Kafitz K. W. Rose C. R. (2008). Sodium signals in cerebellar Purkinje neurons and Bergmann glial cells evoked by glutamatergic synaptic transmission. *Glia* 56 1138–1149. 10.1002/glia.20685 18442095

[B6] Bernardinelli Y. Magistretti P. J. Chatton J.-Y. (2004). Astrocytes generate Na^+^-mediated metabolic waves. *Proc. Natl. Acad. Sci. U.S.A.* 101 14937–14942. 10.1073/pnas.0405315101 15466714 PMC522032

[B7] Berridge M. J. Bootman M. D. Roderick H. L. (2003). Calcium: calcium signalling: dynamics, homeostasis and remodelling. *Nat. Rev. Mol. Cell Biol.* 4 517–529. 10.1038/nrm1155 12838335

[B8] Bordey A. Sontheimer H. (1998). Electrophysiological properties of human astrocytic tumor cells in situ: enigma of spiking glial cells. *J. Neurophysiol.* 79 2782–2793. 9582244 10.1152/jn.1998.79.5.2782

[B9] Carreira B. P. Morte M. I. Santos A. I. Lourenço A. S. Ambrósio A. F. Carvalho C. M. (2014). Nitric oxide from inflammatory origin impairs neural stem cell proliferation by inhibiting epidermal growth factor receptor signaling. *Front. Cell. Neurosci.* 8:343. 10.3389/fncel.2014.00343 25389386 PMC4211408

[B10] Cornell-Bell A. Finkbeiner S. Cooper M. Smith S. (1990). Glutamate induces calcium waves in cultured astrocytes: long-range glial signaling. *Science* 247 470–473. 10.1126/science.1967852 1967852

[B11] Cornell-bell A. H. Finkbeiner S. M. Cooper M. S. Smith S. J. (1990). Glutamate induces calcium waves in cultured astrocytes: long-range glial signaling. *Science* 247 470–473. 1967852 10.1126/science.1967852

[B12] Dallérac G. Chever O. Rouach N. (2013). How do astrocytes shape synaptic transmission? Insights from electrophysiology. *Front. Cell. Neurosci.* 7:159. 10.3389/fncel.2013.00159 24101894 PMC3787198

[B13] Di Capite J. Ng S. W. Parekh A. B. (2009). Decoding of cytoplasmic Ca^2+^ oscillations through the spatial signature drives gene expression. *Curr. Biol.* 19 853–858. 10.1016/j.cub.2009.03.063 19375314

[B14] Fleischer W. Theiss S. Slotta J. Holland C. Schnitzler A. (2015). High-frequency voltage oscillations in cultured astrocytes. *Physiol. Rep.* 3:e12400. 10.14814/phy2.12400 25969464 PMC4463829

[B15] Kirischuk S. Kettenmann H. Verkhratsky A. (2007). Membrane currents and cytoplasmic sodium transients generated by glutamate transport in Bergmann glial cells. *Pflugers Arch.* 454 245–252. 10.1007/s00424-007-0207-5 17273865

[B16] Kuga N. Sasaki T. Takahara Y. Matsuki N. Ikegaya Y. (2011). Large-scale calcium waves traveling through astrocytic networks in vivo. *J. Neurosci.* 31 2607–2614. 10.1523/JNEUROSCI.5319-10.2011 21325528 PMC6623677

[B17] Langer J. Stephan J. Theis M. Rose C. R. (2012). Gap junctions mediate intercellular spread of sodium between hippocampal astrocytes *in situ*. *Glia* 60 239–252. 10.1002/glia.21259 22025386

[B18] Medeiros M. C. R. Mestre A. Inácio P. Asgarif S. Araújo I. M. Hubbard P. C. (2016). An electrical method to measure low-frequency collective and synchronized cell activity using extracellular electrodes. *Sens. Biosensing Res.* 10 1–8. 10.1016/j.sbsr.2016.06.002

[B19] Micali G. Aquino G. Richards D. M. Endres R. G. (2015). Accurate encoding and decoding by single cells: amplitude versus frequency modulation. *PLOS Comput. Biol.* 11:e1004222. 10.1371/journal.pcbi.1004222 26030820 PMC4452646

[B20] Murphy T. H. Blatter L. A. Wier W. G. Baraban J. M. (1993). Rapid communication between neurons and astrocytes in primary cortical cultures. *J. Neurosci.* 13 2672–2679. 8501531 10.1523/JNEUROSCI.13-06-02672.1993 PMC6576490

[B21] Olsen M. L. Khakh B. S. Skatchkov S. N. Zhou M. Lee C. J. Rouach N. (2015). New insights on astrocyte ion channels: critical for homeostasis and neuron-glia signaling. *J. Neurosci.* 35 13827–13835. 10.1523/JNEUROSCI.2603-15.2015 26468182 PMC4604221

[B22] Pasti L. Volterra A. Pozzan T. Carmignoto G. (1997). Intracellular calcium oscillations in astrocytes: a highly plastic, bidirectional form of communication between neurons and astrocytes in situ. 17 7817–7830. 10.1523/JNEUROSCI.17-20-07817.1997 PMC6793927 9315902

[B23] Poskanzer K. E. Yuste R. (2011). Astrocytic regulation of cortical UP states. *Proc. Natl. Acad. Sci. U.S.A.* 108 18453–18458. 10.1073/pnas.1112378108 22027012 PMC3215035

[B24] Rocha P. R. Medeiros M. C. Kintzel U. Vogt J. Araújo I. M. Mestre A. L. G. (2016). Extracellular electrical recording of pH-triggered bursts in C6 glioma cell populations. *Sci. Adv.* 2:e1600516. 10.1126/sciadv.1600516 28028533 PMC5182051

[B25] Rothen-Rutishauser B. Riesen F. K. Braun A. Günthert M. Wunderli-Allenspach H. (2002). Dynamics of tight and adherens junctions under EGTA treatment. *J. Membr. Biol.* 188 151–162. 10.1007/s00232-001-0182-2 12172640

[B26] Sontheimer H. (1994). Voltage-dependent ion channels in glial cells. *Glia* 11 156–172. 10.1002/glia.440110210 7523291

[B27] Spira M. E. Hai A. (2013). Multi-electrode array technologies for neuroscience and cardiology. *Nat. Nanotechnol.* 8 83–94. 10.1038/nnano.2012.265 23380931

[B28] Srinivasan R. Huang B. S. Venugopal S. Johnston A. D. Chai H. Zeng H. (2015). Ca^2+^ signaling in astrocytes from *Ip3r2^-/-^* mice in brain slices and during startle responses *in vivo*. *Nat. Neurosci.* 18 708–717. 10.1038/nn.4001 25894291 PMC4429056

[B29] Verkhratsky A. Steinhäuser C. (2000). Ion channels in glial cells. *Brain Res. Rev.* 32 380–412. 10.1016/S0165-0173(99)00093-4 10760549

[B30] Zur Nieden R. (2005). The role of metabotropic glutamate receptors for the generation of calcium oscillations in rat hippocampal astrocytes in situ. *Cereb. Cortex* 16 676–687. 10.1093/cercor/bhj013 16079243

